# A New Sign of Scarlet Fever

**Published:** 1911-07-22

**Authors:** John W. Moore

**Affiliations:** 40 Fitzwilliam Square West, Dublin


*To the Editor of* The Hospital.

A NEW SIGN OF SCARLET FEVER.

*To the Editor of* The Hospital.

Sir,—In your impression of The Hospital for July 8, 1911, I find, at page 360, a paragraph headed " A New Sign of Scarlet Fever." But is the sign described by Pastia of Bucharest in the *Presse Medicale* in February, 1911, a " new sign " ?

So far as I know it has been long since recognised and described. Personally I claim no priority for describing it in my " Text-book of the Eruptive and Continued Fevers," published by Fannin and Company, Dublin, in 1892, in the following words : " The rash [of scarlatina] reaches its limits by the second or third day, then fades gradually, leaving persistent blood-coloured (petechial) lines in the folds in front of the elbows, in the axillæ, and popliteal spaces. These streaks may be of use for diagnosis."—Yours faithfully,

John W. Moore, M.D.

40 Fitzwilliam Square West,
Dublin, July 12, 1911.

